# Dapsone Lowers Neutrophil to Lymphocyte Ratio and Mortality in COVID-19 Patients Admitted to the ICU

**DOI:** 10.3390/ijms232415563

**Published:** 2022-12-08

**Authors:** Badar Kanwar, Asif Khattak, Richard E. Kast

**Affiliations:** 1 IIAIGC Study Center, Burlington, VT 05408, USA; 2 Department of Neonatal Intensive Care Unit, Hunt Regional Hospital, Greenville, TX 75401, USA

**Keywords:** ARDS, COVID-19, dapsone, inflammasome, neutrophil-to-lymphocyte ratio

## Abstract

Some physicians use dapsone as part of the standard treatment of severe COVID-19 patients entering the ICU, though some do not. To obtain an indication of whether dapsone is helping or not, we undertook a retrospective chart review of 29 consecutive ICU COVID-19 patients receiving dapsone and 30 not receiving dapsone. As we previously reported, of those given dapsone, 9/29 (30%) died, while of those not given dapsone, 18/30 (60%) died. We looked back on that data set to determine if there might be basic laboratory findings in these patients that might give an indication of a mechanism by which dapsone was acting. We found that the neutrophil-to-lymphocyte ratio decreased in 48% of those given dapsone and in 30% of those not given dapsone. We concluded that dapsone might be lowering that ratio. We then reviewed collected data on neutrophil related inflammation pathways on which dapsone might act as presented here. As this was not a controlled study, many variables prevent drawing any conclusions from this work; a formal, randomized controlled study of dapsone in severe COVID-19 is warranted.

## 1. Introduction

In COVID-19, the most common reason for ICU admission is illness progression to ARDS [1]. Meta-analyses of large studies of mechanically ventilated COVID-19 patients admitted to the ICU gave an estimated case fatality rate of 45% [2]. The neutrophilia with accelerated myelopoiesis commonly seen in severe COVID-19 is essential to the typical overproduction of high levels of IL-1beta, IL-6, IL-10, and other inflammatory mediators that contribute to lung parenchyma destruction [3].

As part of the standard treatment of severe COVID-19 patients entering the ICU, some physicians use dapsone, though some do not [3,4]. See the Rationale section below for details. To obtain an indication of whether dapsone is helping or not, we undertook a retrospective chart review on 29 consecutive ICU COVID-19 patients receiving dapsone and 30 not receiving dapsone. Previously reported results of that retrospective chart review showed 31% (9/29) mortality with dapsone and 60% (18/30) mortality without dapsone [5].

That chart review and use of dapsone in ARDS was based on the extensive body of data on dapsone’s clinical effects of reducing neutrophil-mediated tissue damage in other human diseases, prominently the neutrophilic dermatoses, plus the well-established tissue-destructive role of neutrophils seen in fatal COVID-19 cases as briefly reviewed below [3,6,7,8,9].

To better understand dapsone’s potential effects from that chart review, we went back and looked for evidence from standard laboratory monitoring to see if there were any differences in the dapsone-treated patients that might indicate how dapsone might be lowering COVID-19 mortality. Herein, we reported the markedly lower neutrophil-to-lymphocyte ratio (NLR) in dapsone-treated patients. 

Both the administration of dapsone to COVID-19 patients admitted to the ICU and the retrospective chart review were approved by Hunt Regional Medical Center Review Board, Greenville, Texas, USA. Informed consent was obtained for dapsone use from patients and families, with an explanation of the risks, its unproven status, and of our considerations on why it might help. Patients and families were clear that dapsone is not FDA approved for use in COVID-19. 

Consenting patients received dapsone 100 mg once or twice daily orally, depending on physicians’ choice. Along with dapsone, cimetidine 400 mg three times daily was given to diminish dapsone-related methemoglobinemia [10,11,12]. 

## 2. Rationale

Dapsone was the first of modern antibiotics introduced to clinical practice in the 1940’s. It remains in use worldwide as of this writing in 2022.

As a sulfone it does not cross react in those who are sulfonamide allergic. As an antibiotic, it acts by limiting microbial dihydrofolic acid synthesis as do the sulfonamide antibiotics. It is active in Hansen’s disease, other Mycobacteria, toxoplasmosis, Plasmodia, Pneumocystis, and others [13,14]. As used today in dermatology, dapsone suppresses disease activity in the bullous or neutrophilic dermatoses (bullous pemphigoid, dermatitis herpetiformis, cutaneous lupus, etc.) [13,14].

Dapsone functions to suppress disease activity in the neutrophilic dermatoses specifically by inhibiting the tissue destructive action of normal neutrophils that respond normally to pathological disease-related signaling. That underlying triggering signaling remains unaffected by dapsone [15,16,17]. It was on this basis that dapsone has seen ancillary use in treating various cancers such as glioblastoma and others where neutrophils participate in growth facilitation [18,19,20,21].

A significant tissue destructive element of COVID-19 lung dysfunction is wrought by neutrophils [22,23,24,25]. It was on this basis that dapsone was proposed in 2020 to be part of standard care in ARDS, including that during severe COVID-19 [3,4,5,26]. It was also on this basis that several of the physician staff at Hunt Regional Medical Center decided to offer dapsone alongside standard ICU care to patients and families that understood the risk/benefit and who demanded that we do “anything that might help, even if unproven” [5].

Abnormally increased NLR in peripheral blood is a core indicator and mediator of systemic inflammation of any origin, including in COVID-19 [27,28,29,30,31]. Neutrophils, although essential for defense against infections, also participate in tissue destruction when their activity becomes overly exuberant, as occurs in human pathological inflammatory states for which dapsone is used such as bullous pemphigoid, Behcet’s disease, dermatitis herpetiformis, etc. [14,15,16,17,18,19,20,21,22,23,24]. 

**Figure 1:**
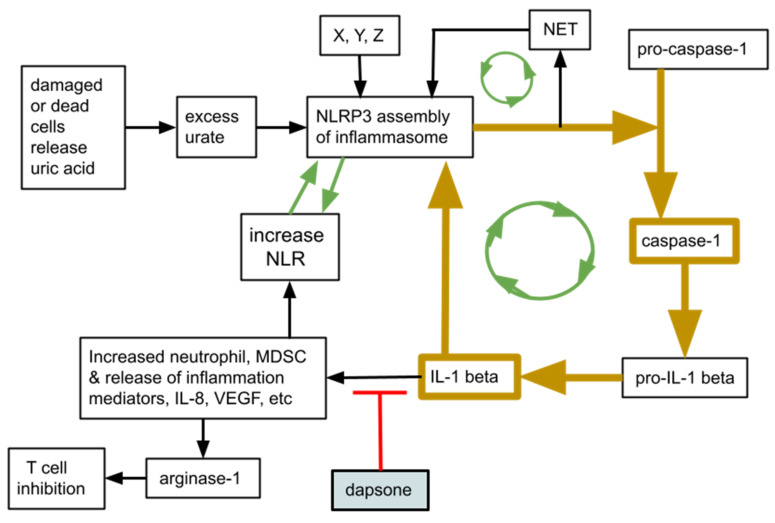
Simplified schematic of the relationship between the neutrophil-to-lymphocyte ratio (NLR), the NLRP3 inflammasome, neutrophil extracellular traps (NET), and IL-1beta. Note the green arrows indicating possible amplifying feedback loops that are one of the elements in “cytokine storm”. There are many other entry points and signaling systems not shown that trigger these elements and many de-amplifying, suppressing elements interacting with this system that are not shown.

Of great significance in severe COVID-19 and any other ARDS, a high NLR is a sign of, and is associated with, increased presence of myeloid derived suppressor cells, (MDSC). MDSC are an immunosuppressive neutrophil (or monocytic) subset that can damage T cells in their vicinity [31,32]. This is diagrammed in Figure 1. Elevated NLR and the emergency granulopoiesis typical of severe COVID-19 are related, and are inextricably linked an increase in MDSC, contributing to the characteristic immunosuppression of ARDS of any origin, including that of COVID-19 [31,33,34].

MDSC numbers and immune suppressive activity increase in direct proportion to the NLR and become an important immunosuppressive element during severe COVID-19 [33,34,35,36,37,38]. Absolute neutrophil numbers and the NLR exist and function within a neutrophil-centered interacting multi-component inflammation system. That system involves circulating neutrophils, tissue resident neutrophils, neutrophil extracellular traps (NET), the NLRP3 inflammasome, caspase-1, and IL-1beta. Figure 1 depicts a simplified schematic of this neutrophil-centered system and the relationship between NLR and several inflammation-amplifying feedback loops between the elements.

Dapsone was shown to reduce IL-1 beta activation [39]. However, the primary rationale for its use in severe COVID-19 was based more on empirical clinical data in its amelioration of neutrophilic dermatoses. The pathophysiologic, neutrophil-centered disease mechanisms active in the neutrophilic dermatoses are also active in generating the ARDS of severe COVID-19 [4,5].

## 3. Patient Population

We retrospectively looked at the NLRs of twenty nine patients entering the ICU with severe COVID-19 who were later given dapsone during their ICU stay. This was compared with a matched group of roughly contemporaneous 30 severe COVID-19 patients entering the ICU for whom the treating physician did not give dapsone. In those receiving dapsone, it was given orally at 100 to 200 mg orally/day with cimetidine 400 mg orally tid. Patient demographics and comorbidities were detailed in the initial report [5]. The two groups were comparable in age, gender, and respiratory status on ICU admission [5]. In all aspects other than dapsone the two groups received the same standard COVID-19 treatment that was also detailed in the initial report [5].

## 4. Results—What Chart Review Revealed

Results from chart review are given in Table 1 and Table 2. In Table 1, lines 1 and 2 show the potentially most important finding—that dapsone use cuts COVID-19 mortality in ICU patients. In ICU patients with severe COVID-19, mortality was 60% without dapsone. When dapsone was added to standard treatment, mortality was 31%.

Table 1, lines 3, 4 indicate that the NLR at ICU entry was roughly comparable in those who received dapsone compared to those who did not. Lines 5, 6, 7, 8 indicate higher NLR in those who went on to die with COVID-19, irrespective of dapsone use or not. 

Lines 9, 10 indicate that those who died on dapsone had a slightly higher entry NLR than did those who survived. Conversely, lines 11, 12 indicate that those who died who did not receive dapsone had a slightly lower entry NLR compared with those who survived without dapsone.

Table 2, lines 13, 14, 15, show data indicating dapsone may be lowering the NLR.

The NLR increased during ICU stay in 52% of those given dapsone but in 70% of those not given dapsone. Correspondingly, NLR decreased in 48% of those given dapsone but in 30% of those not given dapsone.

No case of methemoglobinemia exceeding 11% was seen. There were no cases of untoward effects of dapsone-cimetidine treatment.

## 5. Discussion

In total, 13 studies have been published this year alone (2022) that independently showed that higher NLR in COVID-19 patients entering the hospital was a strong predictor of in-hospital worsening or death [40,41,42,43,44,45,46,47,48,49,50,51,52]. Chart review showed this to be the case also at our ICU, independently of whether dapsone was given or not.

In our study reported here, the NLR increase in 15 of 29 (52%) patients given dapsone compared with 21 of 30 (70%) patients not given dapsone may be significant. This could be due to an inflammation limiting effect of dapsone, or potentially as its functioning as an antibiotic. Our results could reflect some beneficial effect of cimetidine, although it should be noted that all ICU admitted patients were routinely given prophylactic acid suppression treatment. 

The decision to give dapsone was the admitting physician’s choice, so it is possible less ill patients were chosen for dapsone use, although the opposite bias could have been made too. A randomized trial powered to give statistically meaningful results is needed.

As depicted by the green arrows in Figure 1, positive amplification feedback loops are active in severe COVID-19 where overly exuberant granulopoiesis leads to NLRP3 inflammasome assembly that in turn creates further neutrophilia with related tissue damage and MDSC related immune suppression, triggering further NLRP3 inflammasome assembly, etc. [53,54,55]. We conjecture that the specific effect(s) of dapsone on the neutrophils’ contributions to these feedback amplification loops, reflected by the lowered NLR, is responsible for the COVID-19 mortality reduction we saw. 

NLRP3 assembly with consequent activation of caspase-1 and downstream consequences like increased active TNF-alpha, IL-1beta and IL-18 are increased in the lungs of fatal COVID-19 cases [53,56]. Dapsone has been shown to decrease these in a variety of clinical and rodent studies [3,57,58,59,60].

Empirical data presented here may indicate that dapsone diminished the degree of neutrophil-mediated tissue destruction and improved survival in severe COVID-19. Dapsone reduces pathologic tissue destruction in Bechet’s disease, the neutrophilic dermatoses, and in a variety of other pathologies. Our data provide a preliminary indication that we might be able to add COVID-19-related ARDS to that list.

## 6. Conclusions

A chart review of 59 patients with COVID-19 admitted to the ICU indicated that (1) dapsone may lower COVID-19 mortality; that (2) NLR at entry to ICU was roughly comparable for those who did versus those who did not receive dapsone; that (3) collected data tended to confirm what others found regarding higher NLR increasing chance of dying, lower NLR increasing chance of survival; and that (4) our data provide an indication that dapsone might be lowering NLR.

This was not a formal, randomized, controlled trial, so we cannot draw firm conclusions from this limited data set. Given the pronounced reduction in mortality seen in those given dapsone with cimetidine, plus the good safety profile of dapsone and of cimetidine, a formal trial would be warranted.

## Figures and Tables

**Table 1:** NLR data, COVID19 patients admitted to ICU.

1	COVID death rate with dapsone	9/29 = 31%
2	COVID death rate no dapsone	18/30 = 60%
3	entry NLR, all 29 pts before receiving dapsone	av 10.2, median 8.8
4	entry NLR, all 30 pts no dapsone	av 12.7, median 8.1
5	discharge NLR alive, with dapsone, 20/29,	av 8.6, median 5.9
6	discharge NLR dead, with dapsone, 9/29	av 38, median 20
7	discharge NLR alive, no dapsone, 12/30	av 19.8, median 9.6
8	discharge NLR dead, no dapsone, 18/30	av 32, median 30
9	entry NLR, died, with dapsone, 9/29 = 31%	av 13.3, median 10.9
10	entry NLR, survive, with dapsone, 20/29 = 69%	av 8.8, median 8.0
11	entry NLR, died, no dapsone, 18/30 = 60%	av 10.2, median 7.6
12	entry NLR, survive, no dapsone, 12/30 = 40%	av 16.4, median 10.1

**Table 2:** Indications dapsone may lower the NLR during severe COVID-19.

13	NLR increased, # pts, with dapsone	15/29 = 52%
14	NLR decreased, # pts, with dapsone	14/29 = 48%
15	NLR increased # pts, no dapsone	21/30 = 70%
16	NLR decreased # pts, no dapsone	9/30 = 30%

## Data Availability

Any further details are available from the corresponding author, R.E.K.
